# A Study on the Microstructure and Mechanical Properties of Improved 25Ni-20Cr Steel via in Situ Testing

**DOI:** 10.3390/nano15060413

**Published:** 2025-03-07

**Authors:** Penghui Lei, Xiaoyu Ji, Jiahao Chen, Yunhao Huang, Nan Lv, Yulin Fan, Yongchao Hou, Xinsheng Shi, Di Yun

**Affiliations:** 1 School of Nuclear Science and Technology, Xi’an Jiaotong University, Xi’an 710049, China; penghuilei@xjtu.edu.cn (P.L.); 2192112868@stu.xjtu.edu.cn (X.J.); chenjiahao101315@163.com (J.C.); diyun1979@xjtu.edu.cn (D.Y.); 2 State Key Laboratory of Advanced Nuclear Energy Technology, Nuclear Power Institute of China, Chengdu 610041, China; 3 China National Heavy Machinery Research Institute Co., Ltd., Xi’an 710018, China; fylyhx@sina.com (Y.F.); yongchaohou-001@163.com (Y.H.); shizmw123@foxmail.com (X.S.)

**Keywords:** Gen-IV nuclear reactors, N-S35140 austenitic steel, mechanical properties, TEM in situ compressive test, SEM in situ tensile test

## Abstract

To meet the application requirements for structural components in Gen-IV nuclear reactors, it is essential to improve the high-temperature mechanical properties of 25Ni-20Cr (S35140) austenitic stainless steel. In this research, an improved austenitic stainless steel (N-S35140), derived from S35140 steel, was investigated. The scanning transmission electron microscopy (STEM) results indicate that the addition of titanium (Ti) microalloying elements to S35140 steel led to the precipitation of new strengthening nano phases, including M(C, N), MC, MN and Ti(C, N), in N-S35140. These precipitates effectively compensated for the loss of high-temperature strength resulting from the substantial reduction in carbon content. During the in situ transmission electron microscopy (TEM) compressive process at room temperature, the yield strength of N-S35140 steel is 618.4 MPa. At room temperature, the tensile strength of N-S35140 steel is 638.5 MPa, with a yield strength of 392.8 MPa and an elongation of 32.7%, which surpasses those of S35140 steel at room temperature. N-S35140 steel exhibits a tensile strength of 330.6 MPa, a yield strength of 228.2 MPa, and an elongation of 51.4% during the in situ scanning electron microscopy (SEM) tensile test conducted at 650 °C. As a consequence, the improved N-S35140 steel demonstrates significantly enhanced mechanical properties compared to the original S35140 steel, positioning it as a promising candidate for structural components in Gen-IV nuclear reactors.

## 1. Introduction

S35140 (based on Fe-25Ni-20Cr Steel/Alloy 709) austenitic steel is considered as a promising candidate structural material for the components of Gen-IV sodium-cooled fast neutron reactor (SFR) and supercritical water reactor (SCWR), due to its exceptional mechanical properties [1,2,3,4]. The high strength and stability of S35140 steel result from its higher carbon (C) and nickel (Ni) content design, with the primary strengthening phases being (Nb,Ti)CN and M23C6 [5]. Kruizenga et al. [6] reported that S35140 steel exhibited excellent corrosion resistance. However, in Generation IV nuclear reactors, where the operating temperature typically reaches 550 °C or higher [7], the higher carbon content in the S35140 steel negatively impacts its high-temperature aging stability and long-term corrosion resistance. At higher operating temperatures, carbides such as M23C6, M6C tend to precipitate within the matrix and along grain boundaries [8]. The M23C6 phase, in particular, undergoes significant coarsening above 600 °C, which adversely affects high-temperature strength and resistance to supercritical water corrosion [9,10,11]. Meanwhile, in a high-temperature steam environment, chromium (Cr) and oxygen (O) are prone to form volatile chromium hydrides with water vapor, further limiting the working temperature and service environment of austenitic stainless steel [12,13]. Therefore, it is essential to improve and enhance the properties of S35140 austenitic stainless steel to better meet the demanding requirements of high-temperature and corrosive environments in nuclear applications.

The properties of stainless steel in high-temperature service are affected by the content of alloying elements in the material [14,15,16,17]. Compared to the Cr_2_O_3_ film, the Al_2_O_3_ film formed by adding aluminum (Al) to austenitic stainless-steel exhibits superior thermal stability and enhanced corrosive resistance at high temperatures [18,19,20,21]. The addition of elements such as titanium (Ti) and niobium (Nb) improves the oxidation resistance, the corrosion resistance, and the mechanical properties of stainless steel [22,23,24,25,26]. Thus, an improved austenitic stainless steel (N-S35140) was developed based on S35140 austenitic steel using a microalloying design method, with the aim of meeting the application requirements for structural components in Gen-IV nuclear reactors [27]. This microalloying method involved a significant reduction in C content, along with the regulation of microalloying elements such as N and Nb, and the incorporation of controlled amounts of additional elements like Al and Ti. This approach compensates for the loss of high-temperature strength resulting from the significant reduction in C content by promoting the formation of new strengthening phases. Further research is required to comprehensively investigate the microstructure and mechanical properties of N-S35140 steel.

In this paper, the microstructural morphologies and elemental distributions of the N-S35140 are investigated using TEM equipped with Energy-Dispersive X-ray Spectroscopy (EDS) systems [28]. SEM in situ tensile tests and TEM in situ compressive tests were performed to systematically investigate the evolution of the microstructure and mechanical properties of N-S35140 at various service temperatures. Electron backscatter diffraction (EBSD) was used to analysis changes in grain orientation.

## 2. Materials and Methods

The chemical composition of N-S35140 steel is presented in Table 1. In this research, all experimental samples were melted at 1600 °C using a vacuum induction furnace under a vacuum of less than 1 × 10^−4^ Pa. The resulting ingots were forged at a holding temperature of 1200 °C for 2 h, with a final forging temperature of 1100 °C and a forging ratio of 3:1. Following forging, the ingots were held at 1200 °C for 2 h and subsequently rolled with a deformation of 65%. The final rolling temperature was approximately 1050 °C. After rolling, the forgings were quenched by water cooling.

**Figure 1:**
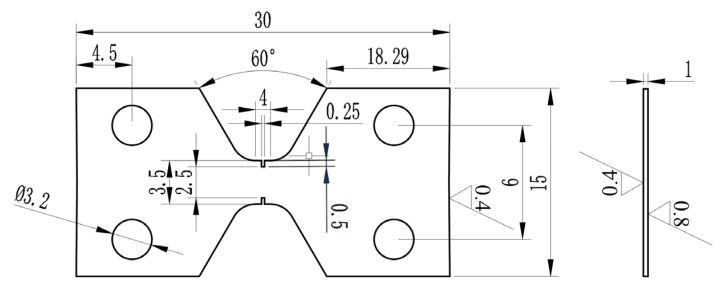
Dimensions of room-temperature tensile specimen (unit: mm).

**Figure 2:**
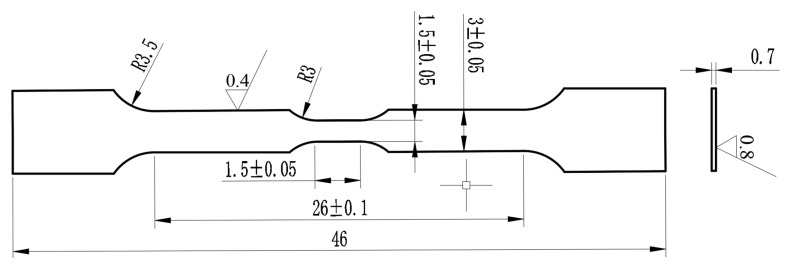
Dimensions of high-temperature tensile specimen (unit: mm).

Grain orientation was analyzed using SEM with EBSD under a vacuum of 5 × 10^−5^ Pa (Gemini 460, Carl Zeiss AG, Oberkochen, Germany). The electrochemical double-spray method was employed to prepare samples for TEM microstructure characterization under a vacuum of 1.1 × 10^−7^ Pa (Talos F200X, Thermo Fisher Scientific, Waltham, MA, USA). The preparation of samples for TEM in situ compressive tests was carried out using a focused ion beam (FIB) under a vacuum of 4.9 × 10^−5^ Pa (Thermo Scientific, Helios 5 UX, Thermo Fisher Scientific, Waltham, MA, USA). The dimensions of the TEM in situ compressive column sample are φ 200 nm × 800 nm. In situ compression experiments were conducted using a PI-95 in situ mechanics sample holder from Bruker. The sample holder was mechanically controlled, piezoceramic, and sensor-controlled in multiple dimensions to ensure precise nanoscale movement of the probes. The probe indentation was controlled by displacement, with a maximum compression displacement of 300 nm and a displacement rate of 10 nm/s. The loading sequence followed a function of 30 s for loading, 10 s for retention, and 30 s for unloading. Room-temperature and high-temperature in situ tensile tests of N-S35140 steel were carried out using an SEM in situ tensile platform. The tensile test temperatures were 25 °C (room temperature), 350 °C, and 650 °C. The heating time to the target temperature was 2–3 h, followed by a 1 h holding period. The dimensions of the in situ room temperature tensile samples are shown in Figure 1, while the dimensions of the in situ high-temperature tensile samples are illustrated in Figure 2. Wire-cutting and electropolishing methods were used to prepare the samples for SEM in situ testing. The carrier platform used for the SEM in situ tensile tests was mechanically controlled in a constant step mode for sample stretching. The room-temperature displacement speed was set at 100 μm/min. For high-temperature tests at 350 °C and 650 °C, the displacement speeds were 60 μm/min and 30 μm/min, respectively. The yield strength, tensile strength, and elongation of the tensile specimens were determined using an extensometer.

## 3. Results and Discussion

### 3.1. Microstructures Characterization

**Figure 3:**
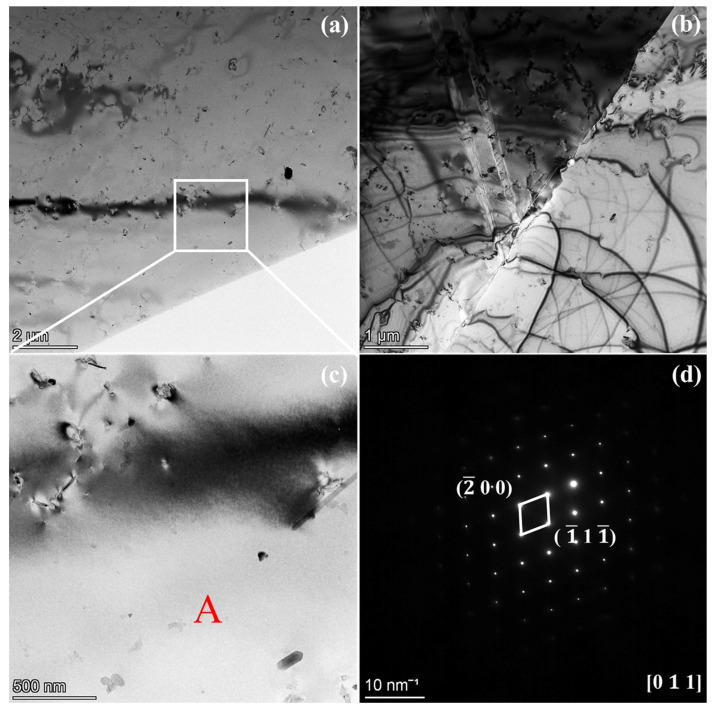
The microstructure morphologies of N-S35140 steel (**a**–**c**); (**d**) SAED of grain A.

The microstructure morphologies of N-S35140 steel are presented in Figure 3. Numerous nanoscale precipitates with distinct interfaces to the matrix grains are visible within the matrix, as shown in Figure 3a. A significant number of precipitates are observed both within the grains and along the grain boundaries, while twin boundaries containing parallel dislocations are evident within the crystals in Figure 3b. The precipitates with different morphologies are observed in Figure 3c. Selected-area electron diffraction (SAED) of grain A, shown in Figure 3d, reveals that the matrix grain exhibits a face-centered cubic (FCC) crystal structure, corresponding to the [0 1 1] band-axis diffraction pattern. The diffraction spots closest to the central transmission spot are attributed to the (−2 0 0) and (−1 1 −1) crystal planes.

**Figure 4:**
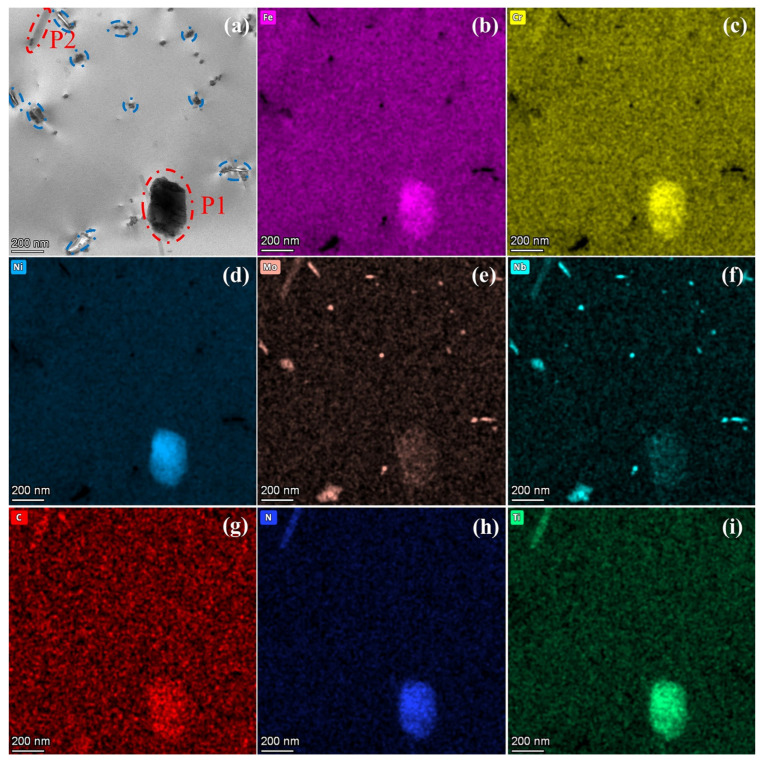
STEM image (**a**) and EDS results (**b**–**i**) of the intragranular structure in N-S35140 steel.

Figure 4 presents the STEM image and EDS mapping results of the intragranular structure in N-S35140 steel. The EDS analysis reveals that precipitate P1 is enriched with elements such as Fe, Cr, Mo, Ni, C, N, and Ti, while precipitate P2 shows elemental enrichment of Cr, Mo, Nb, C, N, and Ti. Both of these precipitates are identified as M(C, N) carbonitrides. The remaining precipitated phases (indicated by blue circle), with sizes ranging from approximately 50 to 100 nm, are enriched in Mo, Nb, and C elements, and are identified as MC-type carbide precipitates. MC-type carbide precipitates are commonly employed to enhance creep resistance at elevated temperatures in advanced heat-resistant austenitic stainless steels [29].

**Figure 5:**
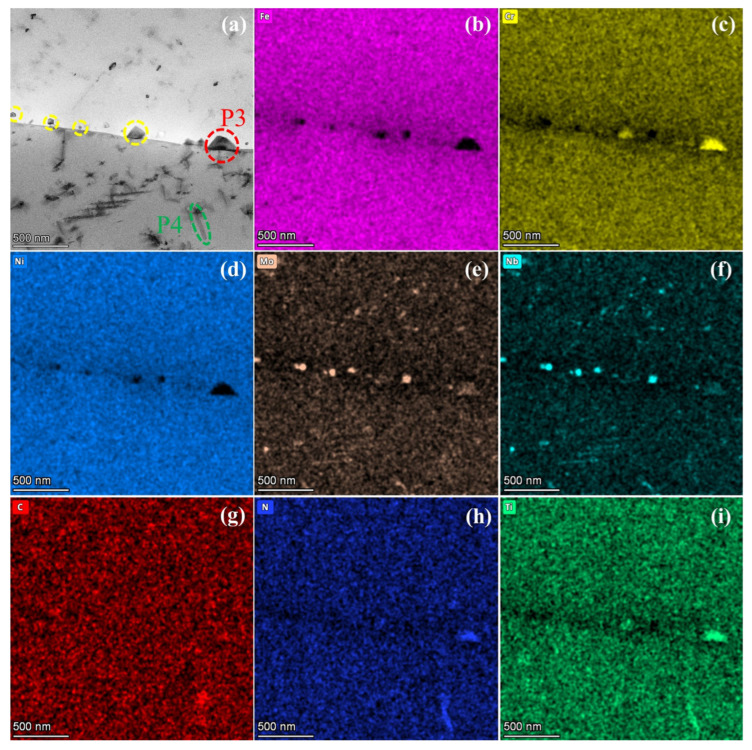
STEM image (**a**) and EDS results (**b**–**i**) of N-S35140 steel near the grain boundary.

The EDS analysis images of S35140 steel near the grain boundary are shown in Figure 5. Figure 5a presents the STEM bright-field image, while Figure 5b–i display the EDS mapping results. The observation indicates that the precipitate P3 is enriched in Cr, N, and Ti elements, corresponding to an MN nitride precipitation phase. The elongated precipitate P4, enriched in C, N, and Ti elements, is identified as the Ti(C, N) carbonitride. The remaining precipitates (indicated by yellow circle), with sizes ranging from 50 to 100 nm, are enriched with Mo, Nb, and C elements, and are identified as MC carbide precipitates. The beneficial trace elements that segregate at the grain boundaries can enhance the grain-boundary bonding by altering the interatomic bonding state [30] and improve strength or hardness by suppressing grain-boundary migration and blocking the dislocation motion [31,32], ultimately leading to enhanced high-temperature strength of the alloy. Therefore, by adding Ti microalloying element, new strengthening phases such as MN and Ti(C, N) were precipitated in N-S35140 steel, effectively compensating for the loss of high-temperature strength resulting from the significant reduction in carbon content. Precipitates at grain boundaries hinder grain-boundary migration, thereby delaying matrix-softening processes, which positively contributes to improved creep resistance [33].

### 3.2. Compression Properties

**Figure 6:**
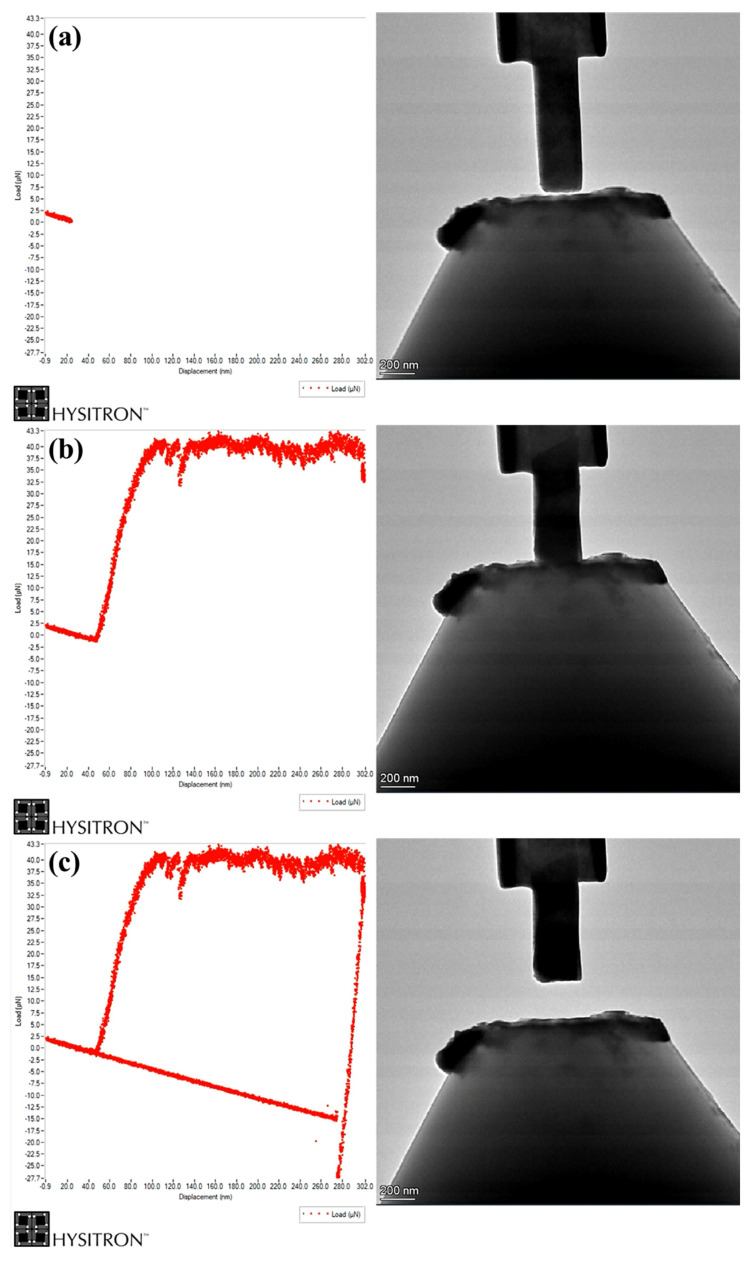
TEM in situ compression process of N-S35140 steel nano pillar: (**a**) starting loading stage; (**b**) compression process stage; (**c**) unloading stage.

The TEM in situ compression process of N-S35140 steel at room temperature is shown in Figure 6. Specifically, Figure 6a–c presents the load–displacement curves and TEM images corresponding to the starting loading, compression, and unloading stages, respectively. At an indenter displacement of 48 nm, the indenter fully contacts the nano pillar, with a maximum load of 43 μN. The plastic flow of the N-S35140 steel nano pillar remained stable throughout the deformation process, with uniform deformation. No significant abrupt changes in displacement were observed.

**Figure 7:**
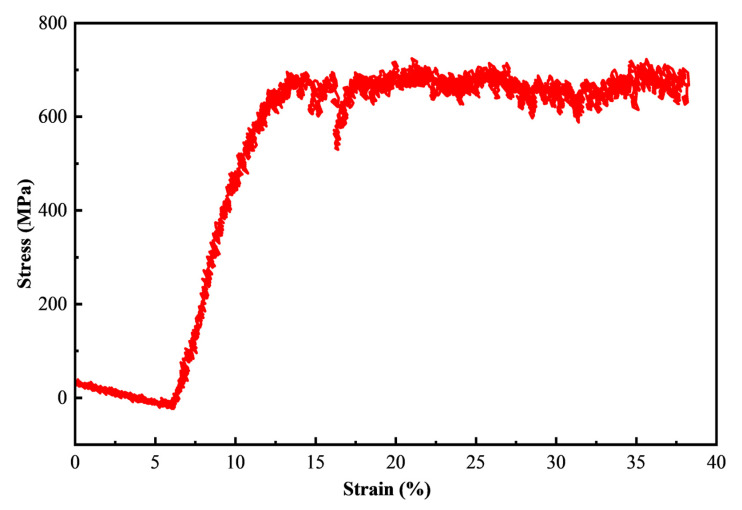
The stress–strain curves of N-S35140 steel during TEM in situ compression at room temperature.

The stress–strain curves of N-S35140 steel during TEM in situ compression at room temperature is displayed in Figure 7. The compressive yield strength is determined to be 618.4 MPa, based on the intersection of the tangent lines of the elastic and plastic deformation regions, which is significantly higher than the compressive yield strength of AISI 304L stainless steel [34]. Low yield strength is often a significant limitation of austenitic stainless steel. However, N-S35140 steel, with its higher compressive yield strength, demonstrates superior shape and dimensional stability. It is worth noting that in the case of TEM in situ testing, the size effect (micro- and nanoscale) is primarily attributed to the reduction in sample size, which leads to a decrease in the number of dislocation sources. This shortage of dislocation sources makes it more difficult to activate dislocation slip for plastic deformation, requiring larger loads to achieve plastic deformation. As a result, the yield strength of N-S35140 steel during TEM in situ compression is higher than that of AISI 304L with classical size due to the size effect [35].

### 3.3. Tensile Properties and Microstructure

**Figure 8:**
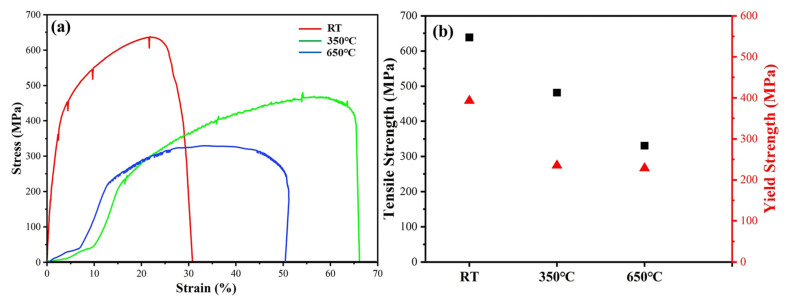
The stress–strain curves (**a**) and tensile strength and yield strength (**b**) of N-S35140 steel during SEM in situ tensile testing at different temperatures.

The stress–strain curves and strength of N-S35140 steel obtained during SEM in situ tensile testing at different temperatures are presented in Figure 8. The size of N-S35140 steel samples for SEM in situ tensile tests is on the millimeter scale, making it suitable for comparison with classical tensile tests. At room temperature, the tensile strength of N-S35140 steel is 638.5 MPa, with a yield strength of 392.8 MPa and an elongation of 32.7%. According to ASTM (A240/A240M-19) [36], the tensile strength, yield strength, and elongation of S35140 steel at room temperature are 620 MPa, 275 MPa, and 30%, respectively. It is evident that the tensile strength, yield strength, and elongation of N-S35140 steel surpass those of S35140 steel at room temperature. During SEM in situ tensile testing at 350 °C, N-S35140 steel exhibits a tensile strength of 481.7 MPa, a yield strength of 234.9 MPa, and an elongation of 66.5%. When the testing temperature is increased to 650 °C, the tensile strength decreases to 330.6 MPa, with a yield strength of 228.2 MPa and an elongation of 51.4%. The yield strength and elongation of N-S35140 steel are superior to those of Alloy 709 steel and AISI 316L at elevated temperatures [37,38], highlighting its enhanced shape and dimensional stability, as well as improved ductility and toughness under load. The segregation of interstitial atoms, such as carbon and nitrogen, in the tensile-stress zone creates a pinning effect on dislocations [39]. As the temperature increases, atomic thermal vibrations intensify, aiding in overcoming this resistance [40]. Consequently, the strength of N-S35140 steel decreases with rising temperature. The serrated curve observed on the stress–strain curve indicates the presence of dynamic strain aging (DSA) at high temperatures.

**Figure 9:**
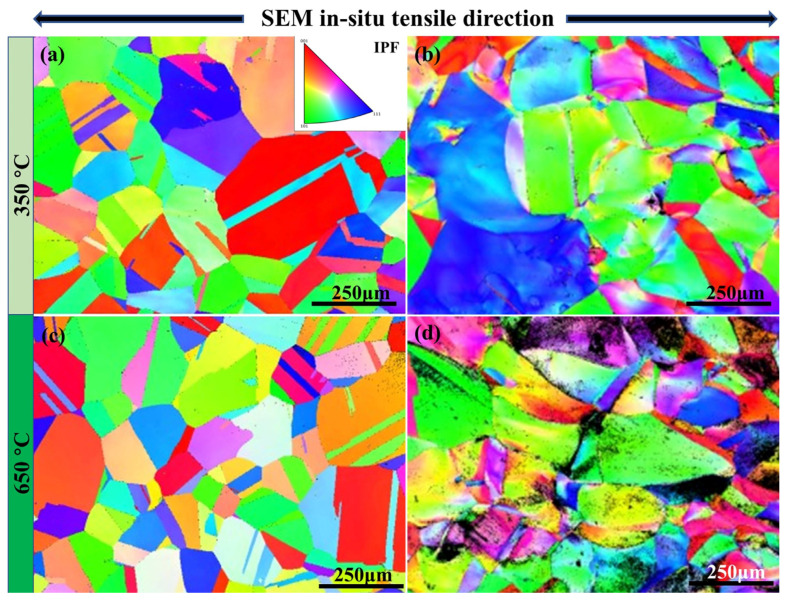
The EBSD results based on inverse pole figure (IPF) map along the *Z*-axis of N-S35140 steel: initial morphologies (**a**,**c**); SEM in situ tensile testing at 350 °C (**b**) and at 650 °C (**d**).

The EBSD result, based on inverse pole figure (IPF) map along the *Z*-axis of N-S35140 steel prior to SEM in situ tensile testing at 350 °C, is shown in Figure 9a. The average grain size of N-S35140 steel is approximately 200 μm. The matrix of N-S35140 steel exhibits an austenitic structure, predominantly consisting of equiaxial crystals before tensile testing. The overall observation reveals a uniform grain distribution and the presence of a twin-crystal structure within some grains, which is primarily attributed to the rolling process of N-S35140 steel. After tensile fracture, the IPF map in Figure 9b shows that the grains have deformed along the tensile direction, with twins still present. Significant grain deformation along the tensile direction is further evidence, shown in Figure 9d, for N-S35140 steel after tensile fracture at 650 °C. Significant grain elongation is observed after deformation, accompanied by an increase in the proportion of grains exhibiting (1 0 1) crystal orientation. Austenitic stainless steels generally have relatively low stacking-fault energy [41], which makes them prone to the formation of stacking faults and twins during tensile deformation. The number of twins decreases significantly after deformation at different temperatures, suggesting that twin-induced plasticity (TWIP) is not the sole contributor to the material’s ductility.

**Figure 10:**
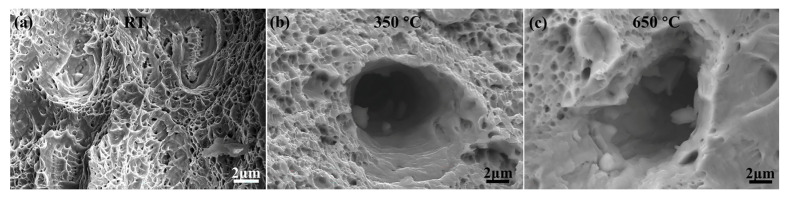
Morphologies of N-S35140 steel during in situ tensile fracture at (**a**) room temperature; (**b**) 350 °C; and (**c**) 650 °C.

The fracture surfaces of N-S35140 steel after SEM in situ tensile testing at room temperature are shown in Figure 10a. Numerous small dimples are observed, indicating a typical ductile fracture mechanism. As the testing temperature increases to 350 °C (Figure 10b) and 650 °C (Figure 10c), the dimples become larger, deeper, and less densely distributed, reflecting the influence of elevated temperatures on the fracture morphology (Table 2). The density, size, and depth of dimples are directly related to the plasticity of the material fracture [42]. Materials with poor plasticity tend to form smaller and shallower ductile dimples during fracture, whereas materials with good plasticity produce larger and deeper dimples. Therefore, the observations suggest that N-S35140 steel exhibits better plasticity at high temperatures compared to room temperature, which aligns with the results shown in Figure 8 and Figure 9.

## 4. Conclusions

In this research, the microstructure of the improved N-S35140 steel for nuclear reactors was observed using a microalloying design method, then analyzed and characterized, while the mechanical properties were researched and analyzed. The following main conclusions were obtained:

(1) By the regulation of microalloying elements in S35140 steel, new strengthening phases M(C, N), MC, MN and Ti(C, N) were precipitated, which compensated for the loss of high-temperature strength after a significant reduction in carbon content. Typically, there is a positive effect of the precipitates distributed along the grain boundaries on the mechanical properties of NS steels. Improved N-S35140 steel has numerous precipitated phases distributed both within the grains and at the grain boundaries. In addition, planar twinning dislocations were observed inside the crystals.
(2) The compressive yield strength of N-S35140 steel during TEM in situ compression at room temperature is 618.4 MPa, which is significantly higher than the compressive yield strength of AISI 304L stainless steel. N-S35140 steel, with its higher compressive yield strength, demonstrates superior shape and dimensional stability compared to AISI 304L stainless steel under load. At room temperature, the tensile strength of N-S35140 steel is 638.5 MPa, with a yield strength of 392.8 MPa and an elongation of 32.7%, which surpasses those of S35140 steel at room temperature. During SEM in situ tensile testing at 350 °C, N-S35140 steel exhibits a tensile strength of 481.7 MPa, a yield strength of 234.9 MPa, and an elongation of 66.5%. When the testing temperature is increased to 650 °C, the tensile strength decreases to 330.6 MPa, with a yield strength of 228.2 MPa and an elongation of 51.4%. The yield strength and elongation of N-S35140 steel are superior to those of Alloy 709 steel and AISI 316L at elevated temperatures.
(3) The grains from the IPF map have deformed along the tensile direction, with twins still present after tensile fracture at 350 °C. Significant grain deformation along the tensile direction is further evident after tensile fracture at 650 °C. Numerous small dimples are observed after SEM in situ tensile testing at room temperature, indicating a typical ductile fracture mechanism. As the testing temperature increases to 350 °C (Figure 10b) and 650 °C (Figure 10c), the dimples become larger, deeper, and less densely distributed. N-S35140 steel exhibits better plasticity at high temperatures compared to room temperature.

In general, the results of this study indicate that N-S35140 steel, as a potential structural material for Gen-IV nuclear reactors, exhibits excellent tensile strength, yield strength, and elongation at high temperatures. Long-term aging, corrosion testing, irradiation, and creep properties of N-S35140 steel will be investigated in further work.

## Figures and Tables

**Table 1:** Actual chemical composition of experimental steel (wt. %) [27].

Experimental Steel	Cr	Ni	Nb	Al	Si	C
S35140	20.0~22.0	25.0~27.0	0.25~0.75	-	0.75	0.1
N-S35140	19.59	24.2	0.77	0.02	0.40	0.03
Experimental Steel	Mo	N	Ti	Mn	Fe	
S35140	1.0~2.0	0.08~0.20	-	1.0~3.0	Bal.	
N-S35140	1.69	0.1	0.04	-	Bal.	

**Table 2:** Summary of testing conditions, areal density of dimples, and average dimple diameter of N-S35140 steel.

Testing Temp. (°C)	Average Dimple Diameter	Areal Density of Dimple (×10^11^/m^2^)
TR	540 nm	12.2
350	695 nm	4.05
650	899 nm	3.15

## Data Availability

The data presented in this study are available on request from the corresponding authors.
